# Successful treatment of recalcitrant Sneddon-Wilkinson disease with secukinumab^☆^

**DOI:** 10.1016/j.abd.2025.501178

**Published:** 2025-08-13

**Authors:** Miguel Mansilla-Polo, Daniel Martín-Torregrosa, Vicent Martínez-Cozar, Rafael Botella-Estrada

**Affiliations:** a Department of Dermatology, Hospital Universitario y Politécnico La Fe, Valencia, Spain; b Instituto de Investigación Sanitaria La Fe, Valencia, Spain; c Department of Pathology, Hospital Universitario y Politécnico La Fe, Valencia, Spain; d Department of Medicine, Facultat de Medicina i Odontología, Universitat de València, Valencia, Spain

Dear Editor,

In 1956, Drs. Ian Bruce Sneddon and Darrell Wilkinson first described Subcorneal Pustular Dermatosis (SPD), later named Sneddon-Wilkinson Disease (SWD) in their honor. They described an unusual chronic blistering eruption that could not be classified as dermatitis herpetiformis or pemphigus.^1^ Whether it is a separate entity, or a variant of Generalized Annular Pustular Psoriasis (GAPS) remains controversial. Clinically, it is more common in middle-aged women and is characterized by flares of pustular lesions, often with an annular pattern and predominantly involving the flexures. These lesions usually progress to erosions, crusts or scales, and residual hyperpigmentation. Histopathologically, SWD is characterized by the presence of subcorneal neutrophilic pustules with negative direct and indirect immunofluorescence tests. It is most commonly associated with monoclonal gammopathies, predominantly IgA. Dapsone is the treatment of choice. However, relapses in a chronic course are frequent.^2^ In this article, we present a case of SWD with a satisfactory response to secukinumab, a monoclonal antibody that selectively inhibits Interleukin (IL) 17A.

A 62-year-old man presented with skin lesions of two years' duration. He had previously been treated with topical and systemic corticosteroids, systemic antihistamines, and doxycycline without improvement. On examination (Figure 1, Figure 2), there were generalized multiple flaccid pustules on an erythematous base, eroding into crusted lesions predominantly in the axillary and inguinal folds. There were no other mucocutaneous lesions and no other systemic symptoms. Histopathology (Fig. 3) showed multiple subcorneal neutrophilic pustules and a mixed perivascular and interstitial infiltrate in the superficial dermis. Direct Immunofluorescence (DIF) was negative. Indirect Immunofluorescence (IIF) was negative for antibodies against epidermal intercellular substances and basement membrane antigens. Immunoblotting confirmed these results and was also negative for antibodies against desmocollin 1 and 3. Genetic testing to exclude IL-36 receptor antagonist deficiency (DITRA syndrome) was also negative. Blood and urine analysis and electrophoresis with immunofixation revealed IgA kappa gammopathy of uncertain significance. Treatment was started consecutively with dapsone, methotrexate, and acitretin, with therapeutic failure with all three agents. Finally, treatment with secukinumab at a dose of 300 mg/week for 4 weeks followed by 300 mg/4 weeks of maintenance was chosen, with a complete response achieved after 2 months of treatment and maintained after 18 months of treatment (Fig. 4). No adverse effects were reported, and the patient remained haematologically stable.

**Figure 1:**
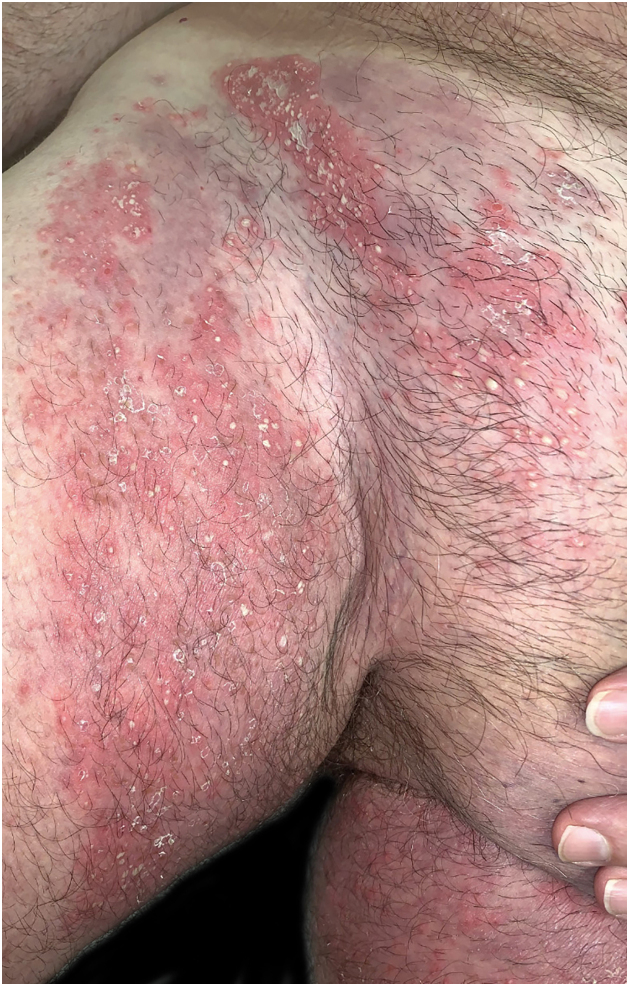
**Clinical presentation of lesions before initiation of secukinumab.** Erythematous papules and plaques with overlying peripheral pustules. The figure depicts lesions in the inguinal folds.

**Figure 2:**
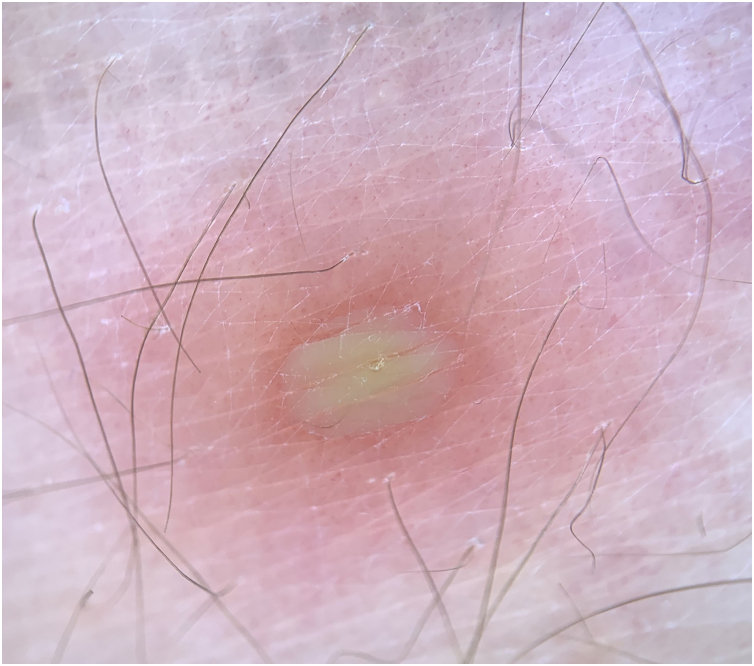
**Dermoscopic presentation of the lesions.** Pustule with a slightly erythematous base.

**Figure 3:**
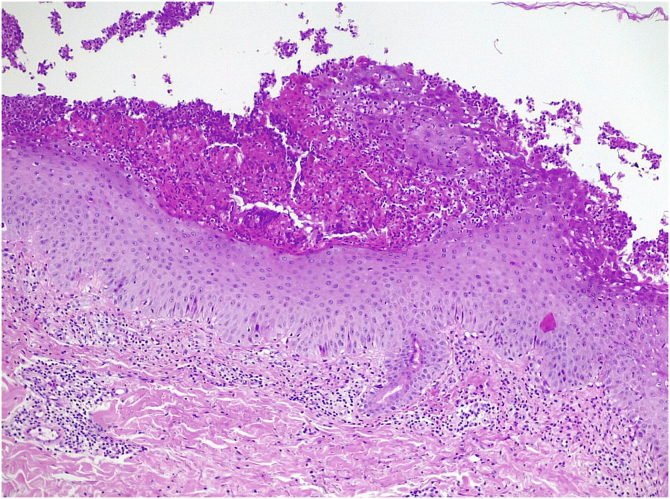
**Histopathological presentation of the lesions (Haematoxylin & eosin 100×).** Moderate perivascular and interstitial inflammatory infiltrate in the dermis, predominantly polymorphonuclear, and in the epidermis minimal spongiosis, parakeratosis and clusters of polymorphonuclear leukocytes forming subcorneal microabscesses. There were no bacterial colonies, acantholysis, papillary atrophy, suprapapillary atrophy or confluent parakeratosis.

**Figure 4:**
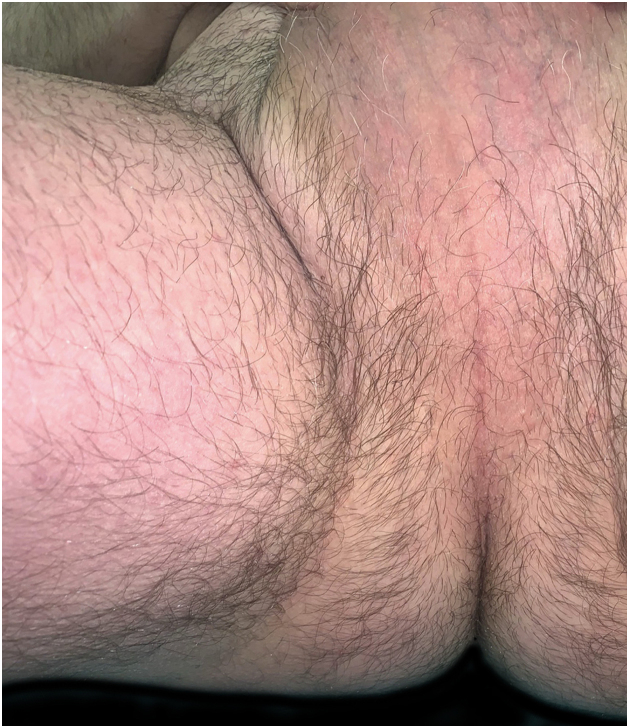
**Clinical presentation of the lesions after 18 months of secukinumab treatment.** Complete resolution of the lesions. The figure depicts lesions in the inguinal folds.

SWD can be challenging both diagnostically and therapeutically. For some authors, it represents a variant of GAPS, while for others it is a separate entity based on its characteristic predilection for folds, non-involvement of nails, palms, soles, and hair, and histological findings with less psoriasiform hyperplasia, fewer tortuous telangiectasias in the dermal papilla and less mitotic figures. However, it must be distinguished from SPD-type IgA pemphigus, which is clinically and histologically identical, although DIF studies show IgA immunoreactivity to desmocollin 1, a desmosomal component of keratinocytes, at the subcorneal level. The presence of anti-desmocollin 1 IgA antibodies is also demonstrated in SPD-type IgA, by using Enzyme-Linked Immunosorbent Assay (ELISA), IIF, or immunoblotting. It must also be distinguished from DITRA syndrome, a rare autoinflammatory disorder caused by mutations in the IL36RN gene, which also causes generalized pustular psoriatic lesions. The classic treatment in SWD, which generally produces a good response, is dapsone.^2^ However, sometimes other treatments must be used because of treatment failure or side effects. In recent years, favorable results have been reported after treatment with anti-TNF agents (adalimumab, etanercept, infliximab), IL-23 inhibitors (guselkumab), phosphodiesterase-4 inhibitors (apremilast, roflumilast) and, more recently, secukinumab.^3^, ^4^, ^5^ We postulate that the response to this drug in our patient with SWD is explained by the role of IL-17A in neutrophil chemotaxis and the demonstrated efficacy of anti-IL-17 agents in pustular psoriasis, an entity closely related to SWD.^5^ In conclusion, we present a case of refractory SWD with a satisfactory response to secukinumab. IL-17 inhibitors may be promising therapeutic agents for the treatment of patients with recalcitrant SWD.

## Research data availability

Does not apply.

## Scientific Associate Editor

Hiram Larangeira de Almeida Jr.

## Financial support

None declared.

## Authors’ contributions

Miguel Mansilla-Polo: Managed clinical treatment and procedures, and wrote the initial version of the article.

Daniel Martín-Torregrosa: Managed clinical treatment and procedures, and wrote the initial version of the article.

Vicent Martínez-Cozar: Managed the pathological study.

Rafael Botella-Estrada: Supervised the work.

## Conflicts of interest

None declared.
